# The Slavcleft: a three-center study of the outcome of treatment of cleft lip and palate. Nasolabial appearance

**DOI:** 10.7717/peerj.10631

**Published:** 2021-02-09

**Authors:** Adam Stebel, Wanda Urbanová, Irena Klimova, Andrzej Brudnicki, Ivana Dubovska, Petra Polackova, Daniela Kroupová, Magdalena Koťová, Piotr S. Fudalej

**Affiliations:** 1 Department of Maxillofacial Surgery, F. D. Roosevelt University Hospital, Banska Bystrica, Slovak Republic; 2 Department of Stomatology and Maxillofacial Surgery, Comenius University in Bratislava, Bratislava, Slovak Republic; 3 Department of Orthodontics and Cleft Anomalies, Dental Clinic, 3rd Medical Faculty, Charles University, Faculty Hospital Royal Vineyard, Prague, Czech Republic; 4 Clinic of Plastic and Reconstructive Surgery, Comenius University in Bratislava, Bratislava, Slovak Republic; 5 Department of Pediatric Surgery, Institute of Mother and Child, Warsaw, Poland; 6 Institute of Dentistry and Oral Sciences, Faculty of Medicine and Dentistry, Palacký University Olomouc, Olomouc, Czech Republic; 7 Department of Orthodontics, Jagiellonian University Cracow, Krakow, Poland; 8 Department of Orthodontics and Dentofacial Orthopedics, University of Bern, Bern, Switzerland

**Keywords:** Cleft lip and palate, Nasolabial appearance, Esthetics, Slavs

## Abstract

**Background:**

There is a multitude of protocols of treatment of cleft lip and palate (CLP) worldwide differing in number of operations, surgical techniques, and timings of surgeries. Despite, facial appearance in subjects with CLP is rarely ideal and residual stigmata are easy to notice in many patients irrespective of the protocol. The prospective controlled investigations are optimal for comparing effectiveness of treatment protocols. Because prospective studies are very challenging to perform in CLP field, it is reasonable to retrospectively assess different surgical protocols to identify the promising ones and then to test them in a prospective way.

**Methods:**

Our objective was to assess the nasolabial appearance in a preadolescent Slavic population with unilateral cleft lip and palate (UCLP) by using the 0–200 numeric scale with reference photographs. Patients treated in Warsaw, Poland (*n* = 32), Prague, Czech Republic (*n* = 26) and Bratislava, Slovakia (*n* = 17) were included in this retrospective study. Each cleft center used a unique surgical protocol. Two panels of professional raters (*n* = 7) and laypeople (*n* = 10) scored blindly the nasolabial esthetics on cropped frontal and profile images with cropped reference photograph present on the same slide. Intra- and inter-rater agreement was assessed with Cronbach’s alpha, intraclass correlation coefficients, *t*-tests, and Bland–Altman plots. Inter-group differences were evaluated with one-way ANOVA and regression analysis.

**Results:**

The agreement within and between raters was acceptable. We found that patients treated in Warsaw, Prague, and Bratislava showed comparable nasolabial appearance on frontal and profile photographs when judged by both professional raters (*p* > 0.05) and laypeople (*p* > 0.05). Regression analysis did not identify influence of gender, group (i.e., Warsaw, Prague, and Bratislava), age at lip repair, surgeon, and age at photographic assessment on esthetic outcome (*p* > 0.05).

**Conclusion:**

This study showed that none of the surgical protocols showed superiority to produce good nasolabial appearance.

## Introduction

Many children and teenagers with cleft lip and palate (CLP) are confronted with comments, questions, staring and teasing related to poor facial appearance and/or speech problems. These behaviors can lead to worse self-perception, lower self-esteem and psychological problems in individuals with CLP (Hunt et al., 2005).

One of the goals of treatment of CLP is to improve facial esthetics and function. However, an association between facial appearance and self-perception or satisfaction with treatment is complex. For example, participants of the Eurocleft study who were most satisfied with their treatment did not demonstrate the best esthetic outcome measured objectively by independent judges (Semb et al., 2005). Meyer-Marcotty & Stellzig-Eisenhauer (2009) showed, in turn, that the self-perception of patients affected by CLP did not correlate with objective results or with how others perceived them. Kuijpers et al. (2021) found that objectively rated nasolabial esthetics was weakly associated with the extent of deformation of nasolabial shape. Despite complexity of the relationship between facial appearance and psychosocial well-being, it is widely recognized that assessment of effectiveness of different protocols of treatment of CLP should include evaluation of nasolabial appearance (Brattström et al., 2005; Mercado et al., 2011). Therefore, the main objective of the current part of the Slavcleft is comparison of nasolabial esthetics in patients with complete unilateral cleft lip and palate treated in three cleft centers—*Warsaw*, *Prague*, and *Bratislava*—using different surgical protocols. The H1_0_ hypothesis was that the nasolabial esthetics in all groups was comparable. An additional objective of the study was to compare results of esthetic rating carried out by professionals and laypeople. The H2_0_ hypothesis was that rating of professionals and laypeople was comparable.

## Materials and Methods

### Ethical approval

The institutional bioethics committees of (1) Institute of Mother and Child (IMC), Warsaw, (2) Medical Faculty of Charles University, Prague, and (3) University Hospital, Bratislava approved this investigation. The reference numbers are respectively: (1) 30/2016, (2) EK-VP/O1/0/2020, and (3) EK/O15/2O2O. All subjects writtenly consented to participate in this study.

### Subjects

Nasolabial appearance was evaluated on frontal and profile images of 75 children with complete unilateral cleft lip and palate (UCLP) who were treated in three centers (Warsaw, Prague, and Bratislava) using different surgical protocols. The images were taken between 2000 and 2012 under standardized conditions for each cleft center (i.e., conditions such as the use of the same background or lightning were standardized within the center; conditions between centers were not standardized). Information about the type of camera, lenses and settings was missing. Description of treatment protocols was presented in the 1st part of Slavcleft study—Table 1.

**Table 1:** Summary of treatment protocols in three groups.

Procedure	Warsaw	Prague	Bratislava
Infant orthopedics	no	no	yes (in some patients)
Lip closure	9 months*	7 months	4 months
Palatal closure		36 months	12 months
Alveolar bone grafting	8–11 years	8–11 years	8–11 years

**Note:**

* According to Warsaw one-stage protocol, lip and palate were repaired simultaneously.

In summary, 32 patients from the *Warsaw Cleft Center* affiliated with IMC were treated with a one-stage repair of the cleft. Tennison–Randall technique was used for lip repair. One surgeon performed all operations. The mean age when photographs were taken was 10.9 years (SD = 1.4; range: 7.5–14). Gender proportion was: males 71.4%, females 28.6%.

A total of 26 children from the *Prague Cleft Center* affiliated with Faculty Hospital Royal Vineard underwent a two-stage repair. Millard technique was used for lip repair. Four surgeons performed all operations. The mean age of record taking was 9.9 years (SD = 1.7; range: 6.6–13.5). Gender proportion was: males 65%, females 35%.

A total of 17 children from the *Bratislava Cleft Cente*r affiliated with the Clinic of Plastic and Reconstructive Surgery, Comenius University, were treated with a two-stage repair. Millard technique was used for lip repair. Four surgeons performed all operations. The mean age of record taking was 8.5 years (SD = 1.6; range: 4.9–11.5). Gender proportion was: males 75%, females 25%.

### Methods

**Figure 1:**
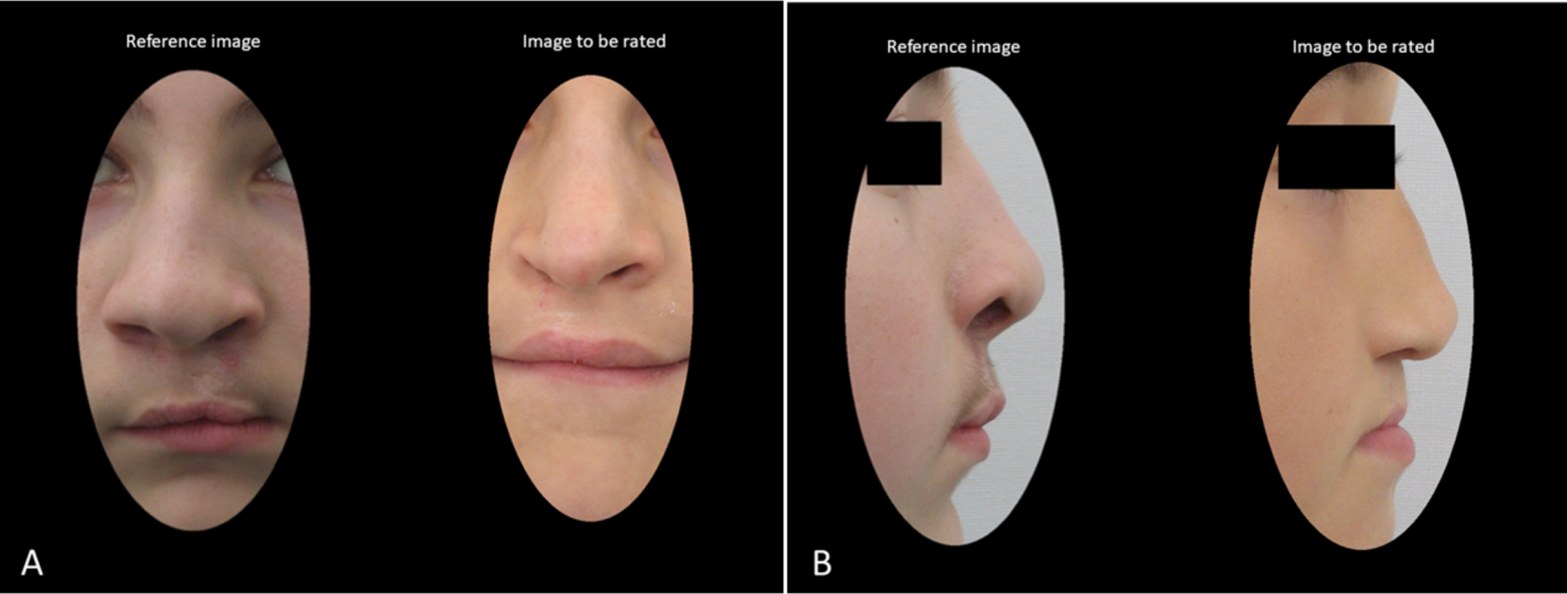
An example of two slides presented during rating session of (A) cropped frontal and (B) cropped profile images with a reference photograph and an image to be rated.

A 0–200 numeric scale with reference photographs, as recommended by Fudalej et al. (2017), was used. Thus, appearance of nasolabial area was rated on cropped frontal and profile images of the affected area with cropped reference photograph of a boy or girl present. The background of the slides was standardized. The images from *Warsaw*, *Prague*, and *Bratislava* were loaded into PowerPoint in a random order for rating. Each slide comprised the frontal (or profile) view of one patient, a reference frontal (or profile) view photograph matched for sex, and a random number assigned (Fig. 1). Reference photographs selected by Fudalej et al. (2017) were used in the current study. The reference photograph had an a priori assigned score of 100. An image to be rated was compared with the reference photograph and scored above 100, if considered more esthetic than the reference, or below 100, if considered less esthetic than the reference.

Seventeen observers (7 professionals and 10 laypeople) rated all photographs. A professional rater was a doctor involved in treatment of CLP. Otherwise a rater was considered lay. All professional raters were orthodontists, pedodontists, and general dentists involved in treatment of patients with the cleft. Of them, 3 were males (age between 26 and 30 years) and 4 were females (age between 25 and 36 years). Professional raters were not involved in the treatment of study participants. Of 10 laypeople, 4 were males (age between 20 and 70 years) and 6 were females (age between 24 and 55 years). Four laypeople were dental technicians and six laypersons had no professional link to medical field. Before rating, a calibration exercise was carried out, so that the raters could familiarize themselves with the rating scale. There was no time limit for rating an image.

In order to assess intra-rater reliability, the 2nd rating session was organized after more than 2 weeks. During the 2nd session, 20 images were rated again.

### Statistical analysis

Subjective assessment of esthetics of facial features produces considerable variation among raters, that is, individual scores can be quite different between raters. To reduce variability the scores for the professionals and the scores for laypeople can be averaged provided there is sufficient coherence among the observers. High coherence means that ranking of images is similar among observers despite variability of individuals scores. Cronbach’s alpha reliability coefficients and intra-class correlation coefficients (ICCs) were calculated to evaluate inter-rater coherence (agreement). If an inter-observer coherence was adequate, the mean scores of observers were used in the “Result” section.

Intra-rater agreement was assessed with ICCs, paired *t*-tests, and Bland–Altman plots (Bland & Altman, 1986). Comparisons among results achieved in 3 cleft centers were carried out with one-way ANOVA tests. Additionally, regression models with gender, group, age at lip repair, age when photograph was taken, and surgeon as independent variables and esthetic score as dependent variable. Four models were made, for esthetic score obtained on frontal and profile photograph and obtained by professionals and laypeople. Statistical significance was established for *p* < 0.05.

## Results

### Reliability

**Figure 2:**
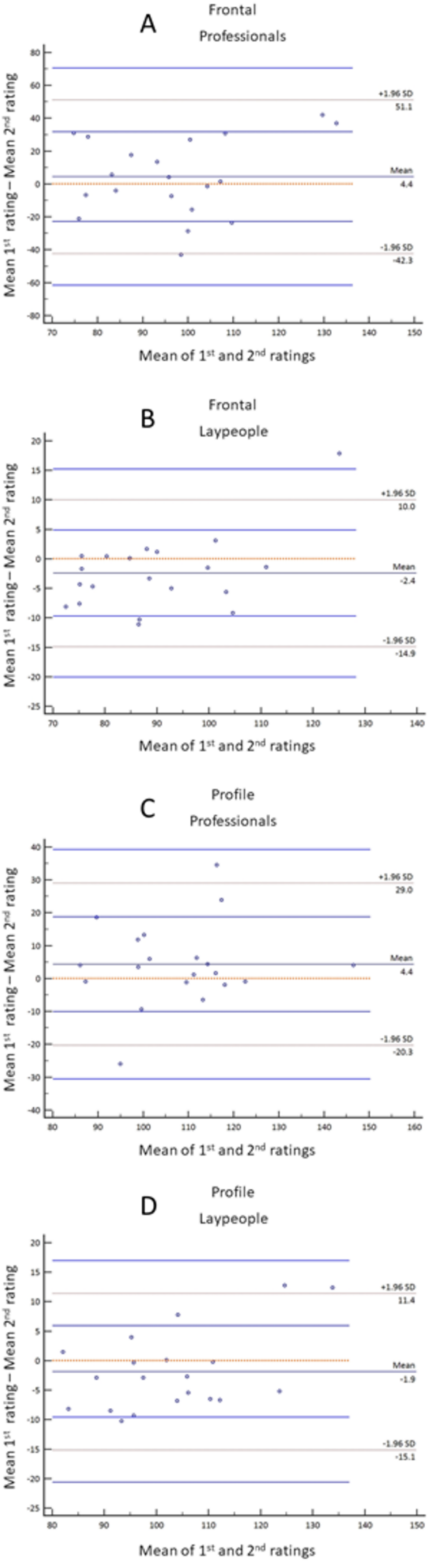
Bland–Altman plots for intra-rater agreement for frontal images rated by professionals (A) and laypeople (B) and profile images rated by professionals (C) and laypeople (D).

Values of Cronbach’s alpha coefficients and ICCs (Table 2) indicated good and very good coherence for frontal and profile ratings among the professional raters and laypeople. The ICCs coefficients and Bland–Altman plots (Fig. 2) showed good intra-rater reliability. Thus, mean scores of the professional and lay raters could be presented in the “Result” section. Additionally, Table 3 shows that the mean results of 1st and 2nd ratings were comparable.

**Table 2:** Assessment of inter-rater reliability.

		Cronbach’s alpha		ICC average		ICC single	
		Coefficient	95% CI, LL	Coefficient	95% CI, LL	Coefficient	95% CI, LL
En face	Lay raters	0.911	0.883	0.860	0.796	0.380	0.281
	Professionals	0.883	0.845	0.824	0.737	0.401	0.286
Profile	Lay raters	0.89	0.856	0.809	0.717	0.298	0.202
	Professionals	0.827	0.771	0.733	0.611	0.282	0.183

**Note:**

CI, confidence interval; LL, lower limit.

**Table 3:** Assessment of intra-rater reliability.

		1st rating		2nd rating		Difference	*p* value	95% CI
		Mean	SD	Mean	SD			
En face	Laypeople	88.48	15.89	90.92	12.78	−2.44	0.102	[−0.53 to 5.42]
	Professionals	99.1	22.27	94.67	17.68	4.43	0.416	[−15.59 to 6.72]
Profile	Laypeople	102.04	15.57	103.9	12.38	−1.86	0.234	[−1.31 to 5.03]
	Professionals	109.88	16.27	105.52	14.87	4.36	0.138	[−10.25 to 1.53]

ICC average		ICC single	
Coefficient	95% CI, LL	Coefficient	95% CI, LL
0.942	0.898	0.462	0.316
0.877	0.782	0.339	0.204
0.932	0.88	0.406	0.268
0.838	0.712	0.27	0.15

**Note:**

CI, confidence interval; LL, lower limit.

### Treatment outcome

Table 4 demonstrates the results of evaluation of the esthetics of the nasolabial area in *Warsaw*, *Prague*, and *Bratislava* groups. There were no inter-group differences for frontal and profile views (*p* > 0.1). The professional raters were less critical (i.e., assigned more favorable scores) than laypeople and the difference was statistically significant (*p* = 0.005 and *p* < 0.001, for frontal and profile views, respectively; scores for Warsaw, Prague, and Bratislava groups combined). Regression analysis (Table 5) demonstrates no effect of the gender, cleft center, age at lip repair, age when photographs were taken, and surgeon who repaired the cleft lip on the esthetic outcome.

**Table 4:** Inter-group differences.

		Warsaw (*n* = 32)		Prague (*n* = 26)		Bratislava (*n* = 17)		*p* value	paired differences
		Mean	SD	Mean	SD	Mean	SD		
En face	Lay raters	87.81	15.44	86.43	11.62	82.97	11.81	0.504	–
	Professionals	93.62	20.73	97.12	15.07	89.35	19.18	0.422	–
Profile	Lay raters	101.43	13.07	104.09	17.76	96.88	15.26	0.327	–
	Professionals	112.90	16.46	112.34	16.06	114.78	17.66	0.890	–

**Table 5:** Regression models.

	Coefficient	SE	*p* value	95% CI, LL	95% CI, UL	*p* value of the model	*R*^2^	Adjusted *R*^2^
En face, Professional raters								
gender (1, male; 2, female)	3.46	5.42	0.526	−7.38	14.29	0.886	0.03	−0.05
group (1, Warsaw; 2, Prague; 3, Bratislava)	−0.65	4.12	0.874	−8.89	7.59			
age at lip repair	−2.68	7.40	0.719	−17.49	12.13			
surgeon	0.01	1.23	0.996	−2.46	2.47			
age at photo assessment	1.63	1.58	0.305	−1.53	4.79			
Constans	76.68	23.13	0.002	30.40	122.95			
En face, Laypeople								
gender (1, male; 2, female)	2.53	3.61	0.486	−4.70	9.76	0.731	0.05	−0.04
group (1, Warsaw; 2, Prague; 3, Bratislava)	−3.09	2.75	0.266	−8.58	2.41			
age at lip repair	−2.03	4.94	0.682	−11.91	7.85			
surgeon	0.24	0.82	0.776	−1.41	1.88			
age at photo assessment	0.48	1.05	0.649	−1.63	2.59			
Constans	84.30	15.43	0.000	53.43	115.17			
Profile, Professional raters								
gender (1, male; 2, female)	5.85	4.76	0.224	−3.68	15.38	0.670	0.05	−0.03
group (1, Warsaw; 2, Prague; 3, Bratislava)	1.99	3.62	0.584	−5.25	9.24			
age at lip repair	−3.47	6.51	0.596	−16.49	9.55			
surgeon	−1.34	1.08	0.221	−3.51	0.83			
age at photo assessment	0.8	1.39	0.567	−1.98	3.57			
Constans	101	20.34	0.000	60.31	141.69			
Profile, Laypeople								
gender (1, male; 2, female)	−2.52	4.45	0.573	−11.42	6.38	0.733	0.05	−0.04
group (1, Warsaw; 2, Prague; 3, Bratislava)	−2.08	3.38	0.541	−8.85	4.69			
age at lip repair	−0.3	6.08	0.961	−12.47	11.87			
surgeon	0.79	1.01	0.441	−1.24	2.81			
age at photo assessment	0.85	1.30	0.515	−1.74	3.44			
Constans	97.44	19	0.000	59.42	135.45			

## Discussion

In this part of the Slavcleft study we compared naolabial appearance in subjects treated with different protocols including surgical management of the cleft lip—centers from Warsaw and Prague used Tennison–Randall technique, while the center from Bratislava used Millard technique. Moreover, we requested 2 groups of raters—professionals and laypeople—to assess blindly frontal and profile photographs. We found that the esthetic outcome achieved in *Warsaw*, *Prague*, and *Bratislava* was comparable (the H1_0_ hypothesis was confirmed) despite significant differences in facial morphology (Urbanova et al., 2016) and dental arch relationship (Fudalej et al., 2019). This seeming inconsistency of the results was also found in the Eurocleft and Americleft studies—participating cleft centers differed considerably for morphological and occlusal outcomes while the nasolabial appearance of the patients treated with various therapeutical protocols was less diversified (Shaw et al., 2005; Russell et al., 2011). There could be several explanations for this phenomenon. First, facial morphology and dental arch relationship were directly affected by deficiency in the growth of hard tissues, whereas the effect of skeletal morphology on the appearance of the nasolabial area could have been modified by deformation of soft tissues of the nose and upper lip. Second, previous studies demonstrated that assessment of esthetics is associated with significant variability of the scores inflating standard deviation from the mean in evaluated groups (Bongaarts et al., 2008; Mercado et al., 2011). As a result, large samples are required to identify inter-group differences, possibly higher than these available in the current study. Finally, esthetics of the area affected by the cleft changes with age, particularly during the growth spurt. However, the subjects evaluated in this study were predominantly before the growth spurt. Thus, it is possible that the inter-group differences would be detectable at later age.

In this investigation we used a numeric scale with reference photographs to determine nasolabial esthetics. Our method was not the most commonly used in the research. Instead, most evaluations were carried out with the aid of a 5-point esthetic index developed for the Eurocleft study by Asher-McDade et al. (1991) or VAS scale without reference images (Sharma et al., 2012; Zhu et al., 2016). Mosmuller et al. (2015) suggested that Asher-McDade esthetic index was superior to the other scoring systems. Nevertheless, the study by Fudalej et al. (2017) in which three methods of scoring nasolabial appearance—5-point esthetic index, 100 mm VAS, and 0 to 200 numerical scale with reference photographs—were directly compared, demonstrated that methods using reference images produced more reproducible results than did VAS or the esthetic index.

As mentioned before, 2 groups of raters—professionals and laypeople—judged nasolabial esthetics of all subjects. We requested persons involved in treatment of the orofacial clefts (professionals) and those without experience in therapy of the cleft deformity (laypeople) because previous studies showed the disagreement between scores assigned by professionals and laypeople—the first group was more, equal, or less critical in its judgements than the second group (Zhu, Jayaraman & Khambay, 2016). In the present study professional raters were significantly less critical than laypeople during evaluation of both types of images—frontal and profile ones. Our findings agree with results of Gkantidis et al. (2013) and Eichenberger et al. (2014) but disagree with Offert et al. (2013) and Foo et al. (2013). Although one can only speculate regarding the cause of this disagreement, it should be noted that despite the different level of criticism, both rater groups were in concordance that nasolabial appearance in patients treated in Warsaw, Prague, and Bratislava was comparable.

It is widely accepted that operator’s skill is related with the outcome of surgery. In the CSAG study (Bearn et al., 2001) the surgeons performing primary lip and palate repair were split into high- and low-volume groups, using operations of five infants with UCLP per year as the cut-off point. According to Bearn et al. (2001) five cases with UCLP is equivalent to an annual caseload of 15 new referrals for primary surgery of all cleft types. The findings of CSAG demonstrated that 5-year-olds operated by high-volume surgeons had better speech and nasal appearance. The effect of surgeon on esthetic outcome, however, disappeared in 12-year-olds. In this investigation, one surgeon with >10 years’ experience in cleft surgery and large annual workload (>50 primary cleft surgeries per year) operated all subjects in Warsaw, while several surgeons were involved in operations of patients in Prague and Bratislava. Therefore, it was particularly interesting to explore the relationship between surgeon’s experience and esthetics outcome. We found no effect of surgeon on the nasolabial appearance as demonstrated by regression models. Thus, our results are in agreement with findings of Bearn et al. (2001).

Initially, we planned to explore the relationship between esthetic and morphological outcomes, that is, between nasolabial appearance and morphology of the face. It was logical to assume that deficient growth of the face could lead to impaired appearance. Unfortunately, we were faced with a common problem in the cleft research, namely, inconsistency of record taking. The dates when lateral cephalograms, diagnostic models, and photographs were made significantly differed for so many subjects that it was impossible to carry out this assessment. Moreover, basal (worm’s-eye) view of the nose—helpful in evaluation of nasal symmetry—was available only for minority of participants and was not therefore included in the study. This study has also other limitations such as relatively small sample size and assessment of non-consecutive patients in *Prague* and *Bratislava* groups. Moreover, retrospective study design, commonly met in the cleft research, is associated with higher risk of bias in comparison to prospective studies.

In summary of the whole project, the results of the Slavcleft were similar to the outcomes of the Eurocleft and Americleft—centers did not perform equally well, although the differences were not dramatic. Better performance was not universal, that is, it did not concern all outcomes, but it was limited to morphological (cephalometric and occlusal) ones. Nasolabial appearance was comparable in all groups and this fact can matter much because, after all, acceptable nasolabial esthetics is likely more important for a patient than skeletal or dental morphology. In this short round-up, we would also like to express a certain dissatisfaction because the problems such as inability to collect consecutively operated patients, inconsistency of record taking, or missing precise information on secondary surgeries weakened our conclusions. Despite, it was worth carrying out this inter-center comparison because it allowed *Warsaw*, *Prague*, and *Bratislava* cleft centers to view ones’ results against a background of other participants.

## Conclusions

Within the limitations of this study we conclude that none of the surgical protocols showed superiority to produce good nasolabial appearance.

## Supplemental Information

10.7717/peerj.10631/supp-1 Supplemental Information 1 Raw esthetics scores.

## Additional Information and Declarations

### Data Availability

The following information was supplied regarding data availability: The raw esthetic scores as well as demographic data are available as a Supplemental File.

## References

[ref-1] Asher-McDade C, Roberts C, Shaw WC, Gallager C (1991). Development of a method for rating nasolabial appearance in patients with clefts of the lip and palate. Cleft Palate-Craniofacial Journal.

[ref-2] Bearn D, Mildinhall S, Murphy T, Murray JJ, Sell D, Shaw WC, Williams AC, Sandy JR (2001). Cleft lip and palate care in the United Kingdom--the clinical standards advisory group (CSAG) study. part 4: outcome comparisons, training, and conclusions. Cleft Palate-Craniofacial Journal.

[ref-3] Bland JM, Altman DG (1986). Statistical methods for assessing agreement between two methods of clinical measurement. Lancet.

[ref-4] Bongaarts CA, Prahl-Andersen B, Bronkhorst EM, Spauwen PH, Mulder JW, Vaandrager JM, Kuijpers-Jagtman AM (2008). Effect of infant orthopedics on facial appearance of toddlers with complete unilateral cleft lip and palate (Dutchcleft). Cleft Palate-Craniofacial Journal.

[ref-5] Brattström V, Mølsted K, Prahl-Andersen B, Semb G, Shaw WC (2005). The Eurocleft study: intercenter study of treatment outcome in patients with complete cleft lip and palate. part 2: craniofacial form and nasolabial appearance. Cleft Palate-Craniofacial Journal.

[ref-6] Eichenberger M, Staudt CB, Pandis N, Gnoinski W, Eliades T (2014). Facial attractiveness of patients with unilateral cleft lip and palate and of controls assessed by laypersons and professionals. European Journal of Orthodontics.

[ref-7] Foo P, Sampson W, Roberts R, Jamieson L, David D (2013). Facial aesthetics and perceived need for further treatment among adults with repaired cleft as assessed by cleft team professionals and laypersons. European Journal of Orthodontics.

[ref-8] Fudalej PS, Urbanova W, Klimova I, Dubovska I, Brudnicki A, Polackova P, Kroupova D, Kotova M, Rachwalski M (2019). The slavcleft: a three-center study of the outcome of treatment of cleft lip and palate. Part 2: dental arch relationships. Journal of Cranio-Maxillofacial Surgery.

[ref-9] Fudalej SA, Desmedt D, Bronkhorst E, Fudalej PS (2017). Comparison of three methods of rating nasolabial appearance in cleft lip and palate. Cleft Palate-Craniofacial Journal.

[ref-10] Gkantidis N, Papamanou DA, Christou P, Topouzelis N (2013). Aesthetic outcome of cleft lip and palate treatment: perceptions of patients, families, and health professionals compared to the general public. Journal of Cranio-Maxillofacial Surgery.

[ref-11] Hunt O, Burden D, Hepper P, Johnston C (2005). The psychosocial effects of cleft lip and palate: a systematic review. European Journal of Orthodontics.

[ref-24] Kuijpers MAR, Maal TJJ, Meulstee JW, Carels CEL, Bronkhorst EM, Bergé SJ, Fudalej PS (2021). Nasolabial shape and aesthetics in unilateral cleft lip and palate: an analysis of nasolabial shape using a mean 3D facial template. International Journal of Oral and Maxillofacial Surgery.

[ref-12] Mercado A, Russell K, Hathaway R, Daskalogiannakis J, Sadek H, Long RE, Cohen M, Semb G, Shaw W (2011). The Americleft study: an inter-center study of treatment outcomes for patients with unilateral cleft lip and palate part 4. Nasolabial aesthetics. Cleft Palate-Craniofacial Journal.

[ref-13] Meyer-Marcotty P, Stellzig-Eisenhauer A (2009). Dentofacial self-perception and social perception of adults with unilateral cleft lip and palate. Journal of Orofacial Orthopedics/Fortschritte der Kieferorthopädie.

[ref-14] Mosmuller DG, Bijnen CL, Kramer GJ, Disse MA, Prahl C, Kuik DJ, Niessen FB, Don Griot JP (2015). The Asher-McDade aesthetic index in comparison with two scoring systems in nonsyndromic complete unilateral cleft lip and palate patients. Journal of Craniofacial Surgery.

[ref-15] Offert B, Janiszewska-Olszowska J, Dudkiewicz Z, Brudnicki A, Katsaros C, Fudalej PS (2013). Facial esthetics in children with unilateral cleft lip and palate 3 years after alveolar bonegrafting combined with rhinoplasty between 2 and 4 years of age. Orthodontics & Craniofacial Research.

[ref-17] Russell K, Long RL, Hathaway R, Daskalogiannakis J, Mercado A, Cohen M, Semb G, Shaw WC (2011). The Americleft study: an inter-center study of treatment outcomes for patients with unilateral cleft lip and palate part 5. General discussion and conclusions. Cleft Palate-Craniofacial Journal.

[ref-18] Semb G, Brattström V, Mølsted K, Prahl-Andersen B, Zuurbier P, Rumsey N, Shaw WC (2005). The Eurocleft study: intercenter study of treatment outcome in patients with complete cleft lip and palate. Part 4: relationship among treatment outcome, patient/parent satisfaction, and the burden of care. Cleft Palate-Craniofacial Journal.

[ref-16] Sharma VP, Bella H, Cadier MM, Pigott RW, Goodacre TE, Richard BM (2012). Outcomes in facial aesthetics in cleft lip and palate surgery: a systematic review. Journal of Plastic, Reconstructive & Aesthetic Surgery.

[ref-19] Shaw WC, Brattström V, Mølsted K, Prahl-Andersen B, Roberts C, Semb G (2005). The Eurocleft study: intercenter study of treatment outcome in patients with complete cleft lip and palate. Part 5: discussion and conclusions. Cleft Palate-Craniofacial Journal.

[ref-21] Urbanova W, Klimova I, Brudnicki A, Polackova P, Kroupova D, Dubovska I, Rachwalski M, Fudalej PS (2016). The Slav-cleft: a three-center study of the outcome of treatment of cleft lip and palate. Part 1: craniofacial morphology. Journal of Cranio-Maxillofacial Surgery.

[ref-22] Zhu S, Jayaraman J, Khambay B (2016). Evaluation of facial appearance in patients with cleft lip and palate by laypeople and professionals: a systematic literature review. Cleft Palate-Craniofacial Journal.

[ref-23] Zhu S, Yang Y, Gu M, Khambay B (2016). A comparison of three viewing media for assessing dental arch relationships in patients with unilateral cleft lip and palate. Cleft Palate-Craniofacial Journal.

